# Mapping Salinity Tolerance during *Arabidopsis thaliana* Germination and Seedling Growth

**DOI:** 10.1371/journal.pone.0022832

**Published:** 2011-08-12

**Authors:** Leah DeRose-Wilson, Brandon S. Gaut

**Affiliations:** Department of Ecology and Evolutionary Biology, University of California Irvine, Irvine, California, United States of America; Texas A&M University, United States of America

## Abstract

To characterize and dissect genetic variation for salinity tolerance, we assessed variation in salinity tolerance during germination and seedling growth for a worldwide sample of *Arabidopsis thaliana* accessions. By combining QTL mapping, association mapping and expression data, we identified genomic regions involved in salinity response. Among the worldwide sample, we found germination ability within a moderately saline environment (150 mM NaCl) varied considerable, from >90% among the most tolerant lines to complete inability to germinate among the most susceptible. Our results also demonstrated wide variation in salinity tolerance within *A. thaliana* RIL populations and identified multiple genomic regions that contribute to this variation. These regions contain known candidate genes, but at least four of the regions contain loci not yet associated with salinity tolerance response phenotypes. Our observations suggest *A. thaliana* natural variation may be an underutilized resource for investigating salinity stress response.

## Introduction

Abiotic factors like temperature, drought and salinity are often temporally and spatially heterogeneous. Because plants are sessile and cannot escape environmental challenges, abiotic stresses are likely to cause selective pressures that lead to local adaptation. Indeed, plants have evolved complex physiological mechanisms to respond to abiotic stresses [1]–[3], many of which are shared among different stress factors [4]–[8]. Variation in water use efficiency and response to drought stress within and between populations has been studied for a number of species; however in most of these cases it is not currently feasible to understand the underlying genetics of this variation [9]–[12].

Soil salinity is a particularly pernicious abiotic stress, both because it is an agricultural problem (>20% of arable land worldwide is currently affected by irrigation-induced salinification; [13]–[14]), and because it fundamentally disrupts plant physiology [15]. Salinity stress is commonly separated into two components: ionic stress and osmotic stress [16], [3]. Plants respond to ionic stress by active exclusion of salt ions or by shunting salt ions into storage tissues in order to maintain cellular homeostasis. Osmotic stress arises because the presence of salts (usually Na+) affects a plant's ability to absorb water and thus limits water availability to plant tissues. The plant response to osmotic stress is physiologically similar to the response to drought stress; ultimately both responses lead to the accumulation of osmolytes and other compounds [17]–[20], [6], [8], often via regulation by abscisic acid (ABA) signaling [21], [22]. If the initial response to either ionic or osmotic stress is insufficient, plants have several mechanisms to limit salt damage, including developmental modifications and the production of hormones and anti-oxidative enzymes [23]–[26], [15].

Relatively little is known about the specific genetic mechanisms underlying adaptation to abiotic stresses [27]–[29], [6]. Most efforts to elucidate the molecular basis of salinity tolerance have employed mutagenesis screens in model organisms. These studies have yielded a number of candidate genes and candidate pathways for the evolution of salinity tolerance. One important example is the Na^+^/H^+^ ion antiporter AtNHX1, which has been cloned in *Arabidopsis thaliana* and used to create transgenic tomato (*Solanum lycopersicum*) and canola (*Brassica napus*) lines [30]–[31]. These lines are able to grow in high (200 mM) NaCl, a concentration usually untenable for wild-type plant growth. Other genes and pathways conferring salt tolerance have been identified, such as genes in the salt overly sensitive (or SOS) pathway [32]–[33], genes involved in ABA signaling or synthesis [34]–[36], and other genes with an array of functions [37]–[41].

The large number of genes implicated in salinity tolerance may not be surprising, because many of the traits underlying adaptation to abiotic stress are quantitative and controlled by perhaps hundreds of genes and dozens of genetic pathways [42]–[44]. While several studies have investigated the molecular basis of salinity tolerance in *A. thaliana* [45]–[46], [4], [19], little attention has been paid to measuring naturally occurring quantitative variation in salinity tolerance and other abiotic stresses in this model species (but see: [47]–[50]). One recent exception is the study of the sodium transporter AtHKT1; allelic variation at this locus was found to regulate sodium accumulation along a coastal cline of *A. thaliana* accessions collected across Europe [51]. However, genetic variation correlated with salinity tolerance and other abiotic stresses has been investigated in a number of other species more thoroughly [52]–[53], most notably salinity in *Helianthus* [54]. Still, in most cases candidate loci have not been identified, making it difficult to characterize the genetic basis of abiotic stress tolerance.

Here we assess natural variation for salinity tolerance in *A. thaliana* and utilize both association and QTL mapping to identify candidate genes that could be contributing to variation in salinity tolerance. In part because of the physiological complexity of the salinity response, there have been few studies looking for QTL for this trait in *A. thaliana* (but see [49] and [36]). There has been, however, limited success for detecting QTL for other physiologically complex traits, such as drought [55]–[56], [47], cold stress [57]–[59] and heavy metals [60]. These studies suggest that physiologically complex traits likely contain some large effect alleles [36], [47]. While QTL approaches may be suitable for detecting rare alleles of large effect, the complementary approach of association mapping may provide additional information, because association mapping incorporates a broader range of natural variation than QTL mapping [61]. As a result, association methods may allow identification of alleles of smaller effect as well as finer mapping resolution [61]–[64]. We note, however, that a large-scale association study in *A. thaliana* recently focused on one aspect of salt tolerance (leaf tissue sodium abundance) and identified only one gene (AtHK1) [64].

To date natural variation in *A. thaliana* salinity tolerance during germination and seedling growth has not been broadly surveyed (but see [65] for an extensive survey of salinity tolerance during vegetative growth). Here we survey natural variation in salinity tolerance, focusing on germination rates, seedling survival, and seedling development measured on an association panel of 96 accessions and on two RIL mapping populations. By comparing QTL and association results, we identify candidate regions that may contribute to tolerance. To delimit candidate genes, we turn to comparative gene expression analyses. We show that *A. thaliana* has wide variation in phenotypes relating to salt tolerance, that this variation has a geographic component, and finally that the genetic architecture underlying phenotypic variation consists of several major QTL and manifold genes of smaller effect. Overall, our multifaceted approach contributes to an understanding of the genetic architecture of salinity tolerance in the broader context of natural variation.

## Materials and Methods

### Plant Materials and Plant Growth

We procured *A. thaliana* seeds for association and QTL mapping from the Arabidopsis Biological Resource Center at The Ohio State University, USA. The association panel consisted of 96 accessions that have been used in previous association and diversity studies [66]–[67]. These 96 include pairs of individuals from 25 natural populations as well as a worldwide survey of commonly used stock center accessions. We also acquired seed from the Col×Ler (CS1899; [68]) and Cvi×Ler (CS22000; [69]) recombinant inbred lines (RILs). Ultimately, the Col×Ler and Cvi×Ler mapping populations were represented in our study by 200 and 162 RILs, respectively. In order to mitigate maternal effects based on non-heritable nutrient provisioning, all accessions and RILs used for phenotyping were based on bulked seed samples from at least 10 mothers.

Plants for phenotyping were grown and germinated in growth chamber at 22°C with long-day (16 hour light/8 hour dark) conditions. Lighting was ∼150 uE m-2 sec-1 for a 16-h photoperiod. Seeds were germinated and grown under control and two high salinity (150 mM & 250 mM) treatments. The control treatment employed 60×15 mm petri dishes with 6 mls per dish of standard MS agar germination media adjusted to ph 5.7 with 1% sucrose (http://www.biosci.ohio-state.edu/pcmb/Facilities/abrc/handling.htm). Our high salt treatments supplemented the control media with 150 mM or 250 mM NaCl.

Each phenotypic measure for each accession was based on a sample of at least 90 total seeds, planted in three replicates of 15 seeds over two blocks that were randomized with respect to the location of the 96 accessions. Control trials were done simultaneously in non-supplemented media. One control replicate of 15 seeds was included in each block for each accession, so ultimately the control sample for each accession consisted of 60 seeds (30 seeds in each of the 150 mM and 250 mM treatments). A total of four phenotypic traits were measured for each accession: total percent germination (PG), time to 50% germination (TD_50_), % cotyledons fully emerged (PC) and % seedling survival at 21 days (PS). Here TD50, PC, and PS are all conditional on germination; only those seeds that germinated are included in the total number used to calculate TD_50_ , PC and PS. We considered seeds to be germinated once the radicle had fully emerged from the seed coat. PG was scored at 24-h intervals from the 2nd day after sowing until the score remained unchanged for five consecutive days or until 100% germination was reached up to 21 days after sowing. To normalize germination percentages, we calculated PG scores as a percent of the germination on non-supplemented control media. For PS, seedlings that were yellowed, swollen or in obvious acute distress were not recorded.

### Association Mapping

Association mapping analysis used the most recent release from the *Arabidopsis* Hap Map data available (http://walnut.usc.edu/2010/data/250k-data-version-3.03). These data include over 240,000 SNPs.

The qqnorm function with Shapiro-Wilk's test, as implemented in R, was used to determine whether the phenotype distribution fit to normality [70]. Both the PC and PS phenotypes had negatively skewed distributions, and the data were log transformed. Transformed data were approximately normally distributed (data not shown). A mixed model approach was used to test for statistical associations between phenotypes and genotypes in our mapping panel [71]. Our model followed Yu et al [71] in that we used both a random effect and a fixed effect to model the portion of phenotypic variation explained by population structure within our sample; this approach performs considerably better than using only a fixed effect to correct for population structure [72]. Previously Yu et al. [71] used the Q matrix generated with STRUCTURE to estimate genome-wide population structure; however it has been suggested that a principal components analysis (PCA) can be used to summarize genome-wide patterns of relatedness instead [73]. PCA analyses were performed by EIGENSTRAT [73]; we used the top six principal components for the P matrix. Our model combined PCA assignments (the fixed effect) for populations with kinship estimates (the random effect) to estimate the effect of population structure. Here we consider kinship as a random effect where the individual random deviations from the population mean are constrained by assuming that the (phenotypic) covariance between individuals is proportional to their relative relatedness (or kinship), which is estimated using genome-wide marker data. The model was implemented in the Tassel association mapping software [74]. Individual random deviations from the population mean were constrained by assuming that the (phenotypic) covariance between individuals was proportional to their relative relatedness (or kinship), which was estimated using genome-wide marker data. The vector of phenotypes, y, was modeled as:
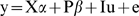
where X contains the genotypes, α is vector of allele effects to be estimated, P is a matrix of population assignments by PCA, β is a vector of population (from PCA assignments) effects, I is an identity matrix, u are the random deviates due to genome-wide relatedness, and e are random deviates due to noise. For each case where a marker had a significant association, a genomic region defined by LD estimates around this marker and the flanking non significant markers was extracted. Genes contained within this genomic region are considered candidates.

### QTL mapping

We used the existing Col×Ler and Cvi×Ler RIL populations, which were genotyped previously [68], [69]. We constructed linkage maps using 243 molecular markers (26, 43, 66, 39 and 69 markers, respectively, for chromosomes 1–5) for the Cvi×Ler mapping population and 352 markers (82, 49, 80, 62 and 79 markers for chromosomes 1–5) for the Col×Ler mapping population. In the Cvi×Ler population, the markers covered 519.5 cM (>85% of the *A. thaliana* genome) and were spaced at intervals ranging from 0.5 to 8 cM, with their average distance being 3 cM. The Col×Ler map spanned 540 cM (∼90% of the *A. thaliana* genome) with markers spaced at intervals from 3–8 cM apart, averaging 4.5 cM between markers. We confirmed marker orders and re-estimated marker position using a joint maximum-likelihood algorithm implemented in the R mapping package R-qtl [75]. We eliminated any markers that were inconsistent in either order or relative position from their locations on the Columbia physical map. Genetic markers that have been anchored to the physical map for Cvi×Ler [76] and Col×Ler were used to estimate the boundaries of confidence intervals. QTL mapping was conducted on RIL means, obtained as the average of 10–12 seeds for each RIL planted in triplicate on three independently randomized blocks for a total of at least 90 observations (10 seeds × 3 pots × 3 blocks), for each RIL. Phenotypic data were transformed to improve normality prior to analyses when necessary.

Heritability (*h* ^2^) was estimated using variance components calculated using the r/kinship package (http://cran.r-project.org/web/packages/kinship/index.html). The R-qtl (http://www.rqtl.org) program implemented in R (http://www.r-project.org), was used for both one and two-dimensional interval mapping in both crosses. We used the EM algorithm and the Haley-Knot regression algorithm at 1-cM steps across the genome. Both algorithms give qualitatively the same results although exact LOD peak locations and confidence intervals varied slightly depending on the mapping algorithm used. In addition to detecting main effects, Rqtl incorporates a multiple QTL model and allows epistatically interacting QTL to be independently identified. In addition a two-way ANOVA was used to test for a significant interaction between specific pairs of QTL. We used 1000 permutations of the dataset for each mapping population to empirically calculate genome wide significance thresholds for LOD scores [77], [78].

### Differential Expression

In order to assess differential gene expression under salinity stress conditions we utilized previously generated gene expression data from the AtGenExpress data set for abiotic stress [79]. This dataset was generated with the Affymetrix ATH1 microarray as part of a comprehensive genome wide expression study in *A. thaliana*. The data were generated in side-by-side experimental series, with three biological replicates for both stressed and control plants. In the original study, plants were subjected to common abiotic stresses, such as temperature, drought, salt, high osmolarity, U-B light and wounding. Here we only consider data from the salinity stress treatment.

We compared expression levels at all seven time points separately, contrasting control and salt treatments at each time point. Expression differences were considered significant for genes classified as up regulated when the signal intensities for both treatment replicates were at least threefold higher than the signal intensities for both control replicates. Similarly, a gene was classified as down regulated when the intensities for both control replicates were at least threefold higher than those of both treatment replicates. By comparing each duplicated control array with its partner for every time point, it was determined that a fold change cut-off of ≥3 results in an average of 0.4% of genes falsely classified as up- or down regulated [79], suggesting our expression criterion are conservative.

## Results

### Natural Variation in Salinity Tolerance

Our four phenotypic traits [% germination (PG), % Cotyledons emergence (PC), Time to 50% germination (TD50) and % Survival (PS); see Methods] were assayed in 96 *A. thaliana* accessions under control conditions, without supplementation of NaCl. PG was >90% for 87 accessions, an additional 7 accessions had PG>85%, and the remaining two accessions had germination rates of 75% and 45%. Once germinated, all lines had PC and PS values >95% after 21 days. In control treatments TD50 means were 4.5 days and ranged from 2–8 days.

**Figure 1:**
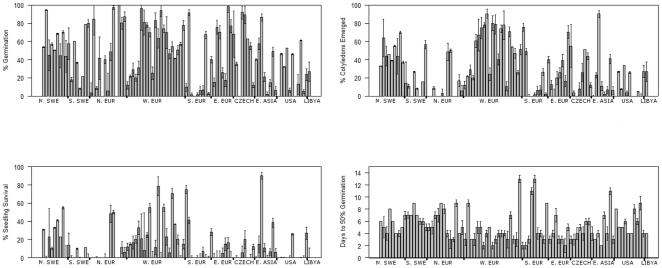
Phenotypes measured in 150 mM NaCl for 96 accessions. The percentage of germination, cotyledon emergence and total seedling survival were measured, as well as the number of days to 50% germination. Days to 50% germination totals included only successfully germinating seeds. Means are based on a total of 300 seeds in 3 replicates. Error bars are the standard deviation. Accessions are grouped loosely by geographic origin: Northern Sweden (N. SWE), Southern Sweden (S. SWE), Northern Europe (N. EUR), Western Europe (W. EUR), Southern Europe (S. EUR), the former Czechoslovakia (CZECH), the United States (USA) and a single accession from LIBYA> The accessions and countries of origin are listed in Figure 2.

We also measured the four phenotypic traits in the presence of NaCl supplemented at 150 mM and 250 mM concentrations. PG varied substantially among the 96 lines on both 250 mM NaCl and 150 mM NaCl treatments (Figure 1). PG scores of >90% were obtained for 10 accessions at 150 mM and 2 accessions (Shakdara and Bay-0) at 250 mM. However, six accessions did not germinate in the 150 mM NaCl treatment, and 13 accessions did not germinate in the 250 mM treatment. An additional 18 accessions had PG values of less than 10% in the 250 mM treatment (data not shown).

PC scores, pertaining to the percent of cotyledons fully emerged after 21 days, also varied dramatically among lines. About 10% of accessions had PC scores >75%; 34% had PC values between 50% and 20%; and ∼10% of accessions that germinated did not advance past initial radicle emergence. PS, survival at 21 days was even more sporadic, with >50% of accessions having survival rates of less than 20%. 32 accessions had no surviving seedlings after 21 days, while only 8 accessions had PS>50%. TD50, time to 50% germination, varied from 2–13 days, with a median of 4 days and a mean of 5.2 days. 85% of accession achieved 50% of total germination within 7 days. Results for 250 mM data were highly correlated with 150 mM data but much sparser and therefore were not used for further analyses.

**Figure 2:**
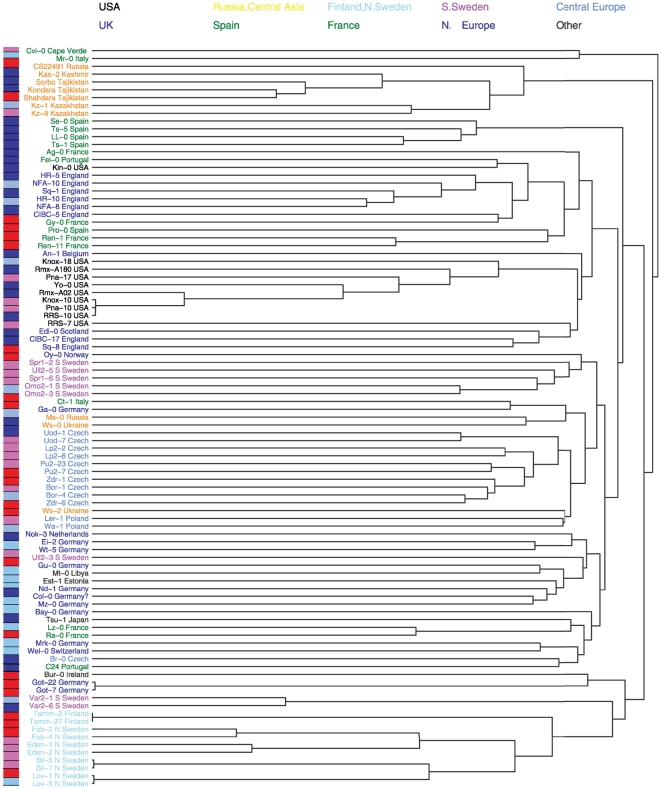
Geographic pattern of salinity tolerance with kinship. The tree on the right shows a hierarchical clustering (UPGMA) of accessions based on pairwise haplotype sharing. Germination (PG) for each accession is mapped on the left with colors indicating relative values for each accession (red indicates greater % germination, blue less germination). Colors for the accessions labels indicate geographic origin. This tree is generated from the same SNP set as used in Atwell et al. [64], and is essentially identical for accessions shared between datasets.

To explore these phenotypes in the context of genetic relationships and geography, we constructed a UPGMA tree with SNP data and mapped PG onto this tree. Variation in salinity tolerance appears to have a geographic pattern, with Western European and Swedish samples having the highest salinity tolerance and Northern and Southern Europe samples having the lowest salinity tolerance (Figure 2). However, although there may be population structure with respect to variation, most geographic regions maintain considerable variation in salinity tolerance (Figure 2).

### Association mapping

**Figure 3:**
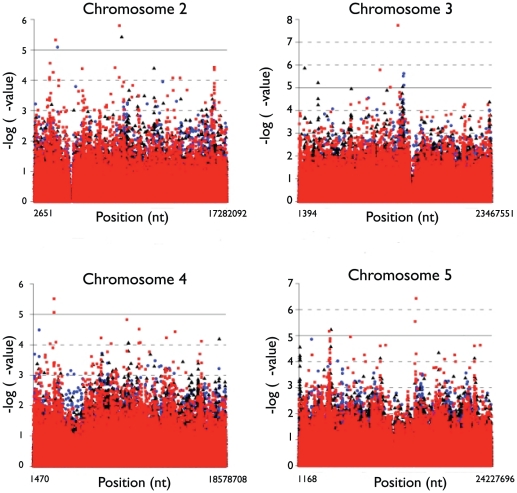
Associations between SNP markers and phenotypes. Peak heights represent negative log *p*-values from tests of association between genotype and phenotype on chromosomes 2–5. PG, PC and TD**_50_** are plotted in blue circles, black triangles and red squares, respectively. The solid grey line marks a significance level of 0.05%.

The PG, PC and TD_50_ phenotypes described above were used for association mapping. We did not include PS because the seedling survival data, with only 64 accessions having any surviving seedling, was too sparse for any confidence in association results. For these analyses we used both genome wide SNP markers and SNPs in candidate genes. Before correction for multiple testing, 780 markers were significant (p<0.05). After correction for multiple testing, 46 moderately significant (p<0.05) markers were detected on chromosomes 2, 3, 4 and 5 across all three phenotypes (Figure 3; Table 1).

**Table 1:** Significant associations for salinity tolerance.

Phenotype^1^	Chrom.	Position (bp)	*p*-value	95%CI (bp)
PG	2	2105258	8.14×10^−06^	2102500–2109450
PG	3	12780819	2.35×10^−06^	12070854–12788125
PC	2	7860901	3.82×10^−06^	7656460–7858000
PC	3	705149	1.39×10^−06^	701156–707860
PC	3	2333725	6.07×10^−06^	2334723–2332597
PC	3	12646834	9.15×10^−06^	12070854–12788125
PC	5	4034766	5.95×10^−06^	3789330–4038700
TD_50_	2	1928602	4.74×10^−06^	1924600–2105258
TD_50_	2	7656460	1.60×10^−06^	7650400–7860901
TD_50_	3	9885343	1.63×10^−06^	9879000–9890050
TD_50_	3	12070854	1.81×10^−08^	12068000–12788125
TD_50_	4	1984900	3.05×10^−06^	1984366–1985678
TD_50_	5	3789330	2.84×10^−06^	3787500–3791430
TD_50_	5	14729553	3.71×10^−07^	14612644–14731417

1 PC = percent germination; PC = percent cotyledons; TD_50_ = time to 50% germination; see text for definitions.

Many of these markers are adjacent and in complete or nearly complete linkage in this set of accessions. In total, association mapping identified ten unique chromosomal regions. Variation in PG was associated with regions on chromosome 2 and chromosome 3. TD_50_ was associated with both of these regions as well as additional regions on chromosomes 2, 4 and 5. Cotyledon emergence (PC) was associated with the same region TD_50_ region (but not the PG region) on chromosome 2, the regions associated with germination and TD_50_ on chromosomes 3 and 5, and two additional regions chromosome 3 (Table 1).

The ten mapped genomic regions contain from 2 to 66 genes per region (data not shown). Three of these regions include obvious gene candidates based on their annotation function (Table 2). These four include AT2G17840, a drought inducible gene that is upregulated in response to salinity stress; AT3G30778, a proline oxidase that is induced by osmotic stress; AT5G12010, an unknown protein that responds to salinity stress, and AT5G38000, an NADP-dependent oxidoreductase involved in response to oxidative stress. In addition to these obvious candidates, these regions include genes involved in ion transport, oxidative stress and several kinases (a class of genes often involved in ionic stress response). For example, the TD_50_ associated regions on chromosome 2 contains CYCB2 a cyclin-dependent protein kinase involved in cell cycle regulation. Similarly, the region on chromosome 3 contains only two genes, an F-box family protein and a zinc finger family protein involved in zinc ion binding. However the primary association maps to a transposable element that contains a binding site for heat shock J family proteins; members of this heat shock family have been implicated in osmotic stress response [80]. Finally, two adjacent regions on chromosome 5 have a suggestive but non-significant association with both TD_50_ and with PC (p = 0.081 and p = 0.06). The first of these regions contains several zinc ion binding proteins, the second region contains adjacent NADP-dependent oxidoreductase genes involved in zinc ion binding and up regulated during general plant stress [79].

**Table 2:** Candidate Genes in QTL and Association regions.

Gene (position)	Chr.	Position of QTL peak (cM) or greatest marker association(bp)	Description	Citation
SFR5 (19.8)	1	20.9	Involved in temperature acclimation and water use	[98]
AT4G19030 (52.1)*	4	52.5	Expression level is reduced by ABA and NaCl.	[99]
SOS2 (70)	5	77.5	Encodes a CBL-interacting protein kinase, responds to NaCl	[33], [100]
ABA3 (25)	1	28.1	Involved in the conversion of ABA-aldehyde to ABA, the last step of abscisic acid (ABA) biosynthesis – salt stress and osmotic stress	[35], [101]
AT3G47950 (64)	3	66	Plasma membrane transporter, mutant is salt susceptible	[102]
RD29A/B (104.7) and ABI2 (112)	5	104.1	involved in temperature stress response and knockouts are salt sensitive	[37], [82], [103]
AT5G05410 (10.5)	5	12.1	Encodes a transcription factor, binds to DRE/CRT cis elements (responsive to drought and low-temperature stress)	[104]
AT2G17840	2	7755605	Drought inducible and upregulated in response to salt stress	[105]
AT5G38000	5	14700552	NADP-dependent oxidoreductase, putative; involved in response to oxidative stress	[106]
AT3G30775	3	12448636	Proline oxidase, induced in response to osmotic stress	[107]
AT5G12010	5	3877812	unknown protein; involved in response to salt stress	[108]

Reported position is that of the most strongly associated marker.

*differentially expressed among salt stress treatments.

### Quantitative trait analyses

The extensive variation in germination response to salinity suggests that NaCl tolerance in *A. thaliana* is a polygenic trait. In order to further investigate the genetic basis of this trait we analyzed two different mapping populations for the same set of phenotypes (PG, PC, PS and TD_50_). The first population was a set of 200 RILs derived from a cross between the Col-4 and Landsberg erecta (Ler-0) accessions [68]. The parental strains had 65% and 28% germination respectively on 150 mM NaCl supplemented media. A second set of 162 RILs derived from a cross of Cvi and Ler, with parental lines having 88% and 24% germination, was also analyzed.

**Figure 4:**
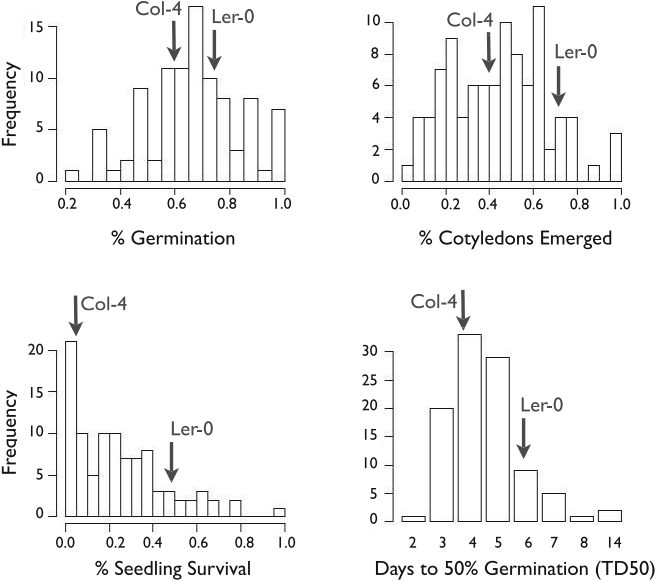
Phenotypic frequencies for the Col × Ler RILs. Phenotypic means for parental lines are indicated by arrows.

In both populations, there was evidence of significant transgressive segregation between parents for all four phenotypes, because multiple RILS are greater than 2 standard deviations (SDs) more extreme than either parent (Figure 4). Transgressive segregation indicates that both parents likely contain alleles contributing to salinity tolerance. Moreover, phenotypic data for all traits measured was highly correlated between 150 mM NaCl treatment assays and 250 mM treatment assays. As the 250 mM data were quite sparse, we considered only the 150 mM treatment data for QTL mapping in both crosses. Finally, estimated heritabilities under 150 mM salinity conditions for the Cvi×Ler cross were *h* ^2^ = 0.91 for PG, *h* ^2^ = 0.71 for PC, *h* ^2^ = 0.76 TD_50_, and *h* ^2^ = 0.57 for PS, (respective heritabilities for the Col×Ler were *h* ^2^ = 0.67, 0.64, 0.71 and 0.36), indicating that the phenotypes measured are heritable.

#### Col × Ler

Across the 200 RILS for this cross, mean PG was 66%, mean PC was 45% and mean PS was 25%. TD_50_ ranged from 2–14 with a mean of 4.5 (Figure 3). There was a significant correlation between PG and PC (*r* = 0.67; p<0.002), but PS was not significantly correlated with either germination phenotypes (data not shown). TD_50_ was also not significantly correlated with other phenotypes.

Using a LOD threshold determined by permutation [78], we identified two major QTL for PG on chromosomes 1 (20.9 cM) and 3 (86.4 cM) that together explained over 30% of variation in PG (Table 3). The QTL on chromosome 3 alone explained >22% of PG variance. Lines containing the Columbia allele at this QTL had a mean germination rate of 72% compared to 55% for those with the Ler allele. The QTL on chromosome 1 had a smaller effect; the Ler allele conferred a moderate increase in germination (60% mean germination for the Col allele; 71% mean germination for the Ler allele). The best germinating lines (>80% germination) have the Col allele on chromosome 3 and the Ler allele on chromosome 1, lines with the opposite allelic series have a mean germination rate of 52%. The QTL on chromosome 1 (20.9 cM) is near the SFR5 gene, which is known to be involved in temperature stress response [81], [35]. ABA3, a gene involved in synthesis of the plant stress hormone ABA, is also nearby (25 cM).

**Table 3:** QTL for salinity tolerance in Col × Ler and Cvi × Ler crosses, with 95% confidence intervals based on bootstrapping.

Phenotype^1^	Cross	Chrom.	Position (cM)	LOD	95%CI (cM)	% variation^2^
PG	Col × Ler	1	20.9	2.02	17–24	8.52
PG	Col × Ler	3	86	3.45	79–89	22.28
PG	Cvi × Ler	1	19	4.13	16.5–21	8.65
PG	Cvi × Ler	4	52.5	2.95	49–56	6.07
PG	Cvi × Ler	5	77.5	5.65	75–79	8.94
PC	Col × Ler	1	28.1	2.54	21–32	9.21
PC	Col × Ler	3	89.4	4.5	82–91	16.89
PC	Cvi × Ler	4	52.5	2.64	47–56	6.72
TD_50_	Col × Ler	3	66	2.62	56–69	8.06
TD_50_	Col × Ler	5	104.1	4.23	102.5–107	13.5
TD_50_	Col × Ler	5	12.1	3.01	74–83	8.5

1 PC = percent germination; PC = percent cotyledons; TD_50_ = time to 50% germination; see text for definitions.

2 % phenotypic variation explained by QTL.

**Figure 5:**
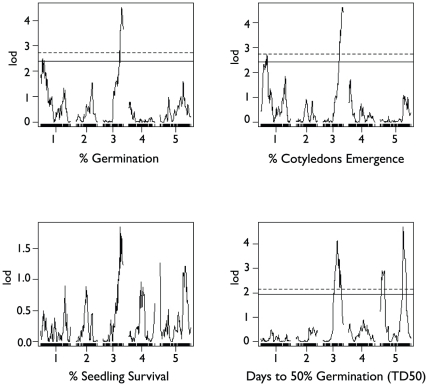
QTL positions with LOD scores for all four phenotypes in the Col × Ler cross. Dashed line indicates the *p*<0.05 significance threshold, and solid line indicates the p<0.10 significance threshold. Significance thresholds are based on 100,000 permutations for phenotypes.

The confidence intervals for QTL contributing to PC overlap two of the QTL for PG. The PC QTL on chromosome 3 maps to a nearly identical location on chromosome 3 (89 cM) (Table 3). However, the other cotyledon QTL maps to a slightly different location on chromosome 1 (28.1 cM) (Figure 5, Table 3), nearer ABA3. Together these QTL in the Col×Ler cross explain ∼26% of PC variation. Similar to PG, the Col allele on chromosome 3 is associated with increased cotyledon emergence, while the Ler allele on chromosome 1 is associated with increased cotyledon emergence.

Three QTL for time to 50% of total germination (TD_50_) were found in the Col×Ler cross: a large-effect QTL at 104.1 cM on chromosome 5, a small effect QTL at 12.1 cM on chromosome 5, and an additional small effect QTL on chromosome 3 (39 cM) (Figure 5). Together these three QTL explain ∼22% of variation in TD_50_ (Table 3). The larger QTL on chromosome 5 is near genes RD29A and RD29B (104.7 cM), which are involved in temperature stress response, drought stress response and confer salt-sensitive knockouts [82, this study, unpublished data]. The other QTL on chromosome 5 is near a member of the Heat Stress Transcription Factor (Hsf) family and is regulated by DREB2A, a transcription factor involved in drought response. The chromosome 3 QTL is not near any known abiotic stress genes. A single small effect QTL for PS was found on chromosome 3 (Table 3). There are no obvious candidate genes at this location but confidence intervals for this QTL overlap those for germination QTL on chromosome 3 (86 cM).

#### Cvi × Ler

The Cvi×Ler cross had a mean PG of 72%, but overall the RILs in this cross were less able to survive in the saline environment than the Col×Ler cross, with mean PC 33% (vs. 45%) and mean PS ∼11% (vs. 25%). TD_50_ ranged from 2–9 days with a mean of 5 days (Figure 3). There was a significant correlation between PG and PC in this cross (r = .71; p<0.003), much like in the Col×Ler cross. PS and TD_50_ were not significantly correlated with other phenotypes, however (data not shown).

In this cross four QTL for PG were mapped to chromosomes 1, 3, 4 and 5 (Table 3, Figure S1). Together these four QTL explain ∼27% of variation in total germination. The Ler alleles for QTL on chromosomes 1 and 4 contributed to increased PG; the Cvi alleles at the remaining two QTL on chromosomes 3 and 5 (39.0 cM and 77.5 cM) contributed to increased total germination. The QTL on chromosome 1 maps to the same location as that in the Col×Ler cross (19 cM), but is a major QTL in this cross and explains ∼10% of the variation. This QTL is significantly associated with PC as well, with QTL for both traits mapping near the SFR5 gene (cM 19.8). Additional QTL on chromosomes 4 (cM 52.5) and 5 (77.5 cM) were mapped for both PG and PC. The QTL on chromosome 4 (52.5 cM) is not near any known candidate genes, but a scan of loci within this region reveals several candidates (Table 2). The chromosome 5 QTL (77.5) is very near SOS2 (70 cM), which is involved in salinity stress response in the salt overly sensitive pathway. The small effect QTL on chromosome 3 (39.4 cM) is not within the confidence intervals for the QTL mapped on chromosome 3 in the Col×Ler cross. However it also is near ABI3 (38 cM), a gene that is responsive to the plant stress hormone ABA and is active in seeds and required for embryo development [34].

### Identifying Candidate Genes with Gene Expression Data

In order to identify potential candidate genes in genomic regions identified by association and QTL mapping, we turned to comparative gene expression data. These data were produced by measuring relative gene expression between control and salt treatments over a time series of seven observations in 36 hours [79]. Out of approximately 24,000 genes on the array, 349 genes were differentially expressed at least 3 fold between control and salinity treatments for at least one time point. We compared these differentially expressed genes to annotated genes from regions identified through both association and QTL analysis. To delimit these regions, we included an arbitrary 1 Mb region around the peak of each inferred QTL, ensuring that we encompassed the 95% confidence regions of all mapped QTL, and 80 kb around the highest scoring association marker. Out of 349 differentially expressed genes, 70 fell within QTL regions, and an additional four lie within association regions (Table S1).

## Discussion

In the past five years there has been rapid growth of studies using natural variation in *Arabidopsis thaliana* to study traits of interest [64], [83]. The motivations for these studies vary from an attempt to exploit natural variation in order to find new genes involved in specific aspects of plant physiology, to trying to understand the molecular basis of adaptations to the local environment. Regardless of the ultimate goal of these studies, the immediate question is usually the same: what is the underlying genetic architecture that affects variation in the trait? This question is unfortunately often difficult to answer. Often even large phenotypic differences are due to variation at multiple loci, and the contribution of any individual locus to the phenotype may be small.

Despite the vast increase in studies using natural variation in *Arabidopsis thaliana* to study traits of interest, variation in abiotic stress phenotypes has generally not been well studied across a broad group of accessions. One exception is a large-scale association study, which investigated several phenotypes that could be related to abiotic stress, including sodium ion content in plant tissue [64]. However, the current work represents the most comprehensive picture of salinity tolerance variation during germination and early seedling development in *Arabidopsis* to date. We have shown that extensive variation exists for salinity tolerance in natural accessions of *A. thaliana* for percent germination (PG), cotyledon emergence (PC), seedling survival (PS) and time to 50% germination (TD_50_).

Not surprisingly, some of these phenotypes are correlated. For example, we found that PG and PC are highly correlated across our sample of 96 accessions (r^2^ = 0.69), but PG and PC were less-strongly correlated with PS (r^2^ = 0.31 and r^2^ = 0.35). In fact, in some accessions germination was <30% but survival for those seeds that germinated was >90%. These results are in agreement with other studies that suggest salt stress during germination and vegetative growth may have different underlying genetic controls. A similar pattern has been documented in soybean (*Glycine max*; [84]), wheat (*Triticum aestivum*; [85]), alfalfa (*Medicago sativa*; [86]), barley [87], and tomato [88]. TD_50_ was slightly positively correlated with the germination phenotypes (PG and PC) in both crosses and natural accessions.

Another feature of phenotypic variation for salt tolerance is that it is somewhat geographically structured (Figure 2). However, this variation differs markedly from other phenotypes that have been studied with the same accessions or with other global *A. thaliana* samples. To the extent we can discriminate with our sample, salt tolerance during germination and seedling growth does not show clinal patterns as have been described in flowering time, drought tolerance, and sodium accumulation phenotypes [50], [51], [89]. A final important feature of phenotypic variation, which was demonstrated previously for one cross [49] and has been repeated here with two, is that phenotypic variance among RILs is larger than either set of parents (Figure 3). This pattern suggests that salinity tolerance in *Arabidopsis* is polygenic, with alleles from both parental lines contributing to both increases and decreases in salinity tolerance.

We have used both association and QTL approaches to identify genome regions that harbor alleles that contribute to salinity tolerance. QTL mapping remains the most direct approach for identifying causal genes, but it often requires considerable effort and may be quite time consuming to isolate a causative gene. More recently association mapping has enabled mapping variation to smaller genomic regions, but confounding factors such as population structure make it difficult to distinguish true associations from false positives. Correction methods to deal with population structure often eliminate true associations [72]. For these reasons it is often of interest to combine multiple lines of evidence when looking for genetic variation underlying a phenotype of interest [83].

We identified ten genomic regions associated with phenotypic variation in our association mapping panel and 8 larger genomic regions in the QTL mapping. As expected the two crosses generated a largely different set of QTL. The QTL regions included six in the Col×Ler cross and three in the Cvi×Ler cross, with one overlapping region between the crosses. The regions identified by association and QTL analysis overlap in six locations, and thus we have identified 10 genomic regions that are associated with phenotypic variation in salinity tolerance. This suggests there are at least 10 loci involved in salinity tolerance in *A. thaliana*, which is a similar number to that suggested from QTL studies in barley [87] and in tomato [88]. Many of the regions we have identified are large and may contain multiple loci affecting salinity tolerance. Moreover, it is likely that we have missed many loci due to statistical power; for example, our association results are certainly underpowered with this set of 96 accessions [67], [72]. Residual population structure may also confound true associations with false positives. For both of these reasons, we are unsure of our ability to map any genomic region confidently with the association approach. The QTL approach does not suffer from this lack of power issue, however it is constrained both by the genetic variation contained with the parents used in the crosses and by our limited ability to map effects to small genomic regions.

Nonetheless, we have identified some interesting candidate genes based on location and gene annotation (Table 2). For example, the QTL on chromosome 1 that was found in both crosses (19–20 cM) is near genes *ABA3*, (24 cM) which encodes a molybdenum cofactor sulfurase involved in the last step of ABA biosynthesis [35], and *SFR5* (19.8 cM) a gene involved in freezing tolerance [81]. ABA3 is interesting because it is ABA plays a role in osmotic stress response during germination [90], [91]. It is known that *aba* and *abi* mutants can sometimes germinate better then wild type parents during salinity stress [92]–[93], [49]. Additional evidence for the involvement of ABA response in salinity tolerance comes from studies in barley supporting a role for ABA-related genes in the control of salt responses during germination [87]. This line of evidence suggests that natural allelic variations in genes involved in ABA signaling or biosynthesis are potential candidates to account for the some of the differences in salt responses seen here. However, a whole suite of other environmental stress response genes colocalize with the mapped regions (*SFR5*, *RD26*, *CBF1*, and several *DREB* family genes; Table 2) might be responsible for the germination differences between strains.

Some of the genes mentioned here as candidates for salinity tolerance are likely to be pleiotropic, affecting multiple different plant phenotypes. For example, there is some evidence from our data that more saline tolerant accessions are also slower to germinate (data not shown). Additionally, because salinity response is physiologically complex, it is likely that salinity tolerance could have similar pleiotropic effects as is seen in drought response, where phenotypes such as development, morphology and growth rate are often affected [94].

We have also examined gene expression data to attempt to verify our candidate genes and our gene regions. Expression studies may provide valuable information by further narrowing the list of candidate genes likely to be causally linked to the trait. Unfortunately, however, no genes have been identified with all three methods – i.e. by association mapping, QTL analyses and gene expression analysis. There is, however, overlap in genes between both mapping approaches and differential expression. Of the 1700 genes in QTL regions and the 215 from our association mapping, 66 overlap but show no evidence of differential expression in saline treatment. However, 71 of the 1700 (4%) genes from the QTL regions are differentially expressed and five of the 215 genes (∼2%) from association mapping overlap with the gene expression data. Of these, only one of our list of ‘strong’ candidates in Table 2 (AT4G19030) is differentially expressed.

There is still considerable debate about the value of comparing differentially expressed genes and mapped genomic locations. There are several issues to consider; first, the genes may not be differentially expressed within a single genotype, but rather between genotypes that differ in salt tolerance, thus we would not see evidence of expression differences within a single accession, as considered in the expression experiment. Second, the expression patterns may differ between tissues; we mapped primarily germination traits, but gene expression was assayed in vegetative tissue. Finally, we may have mapped polymorphic trans-acting regulators of structural genes, and not the genes themselves. If this is true, there is no expectation that mapped regions should coincide with differentially expressed genes. We would expect, however, that further studies of eQTLs would map to the same regions. In other words, although much of the phenotypic variance observed in abiotic stress tolerance is likely due to expression differences, ultimately these expression differences are regulated by genetic changes that can, theoretically, be mapped [95]–[96]. We expect that as more information about interacting gene networks is available, these analyses will provide additional insight not provided by either mapping or expression analysis singularly [96]–[97].

## Supporting Information

Figure S1 **QTL positions with LOD scores for all four phenotypes in the Cvi×Ler cross.** Dashed line indicates the *p*<0.05 significance threshold, and solid line indicates the p<0.10 significance threshold. Significance thresholds are based on 100,000 permutations for phenotypes.
(TIF)

Table S1 **Differentially expressed genes that either fall into QTL regions or are associated with salinity response.** Genes that lie in a QTL region are listed under EXP_QTL; genes identified by association are listed under EXP_ASSC.
(XLS)
